# The combination of recombinant and non-recombinant *Bacillus subtilis* spore display technology for presentation of antigen and adjuvant on single spore

**DOI:** 10.1186/s12934-017-0765-y

**Published:** 2017-09-12

**Authors:** Wojciech Potocki, Alessandro Negri, Grażyna Peszyńska-Sularz, Krzysztof Hinc, Michał Obuchowski, Adam Iwanicki

**Affiliations:** 1 Department of Medical Biotechnology, Intercollegiate Faculty of Biotechnology UG-MUG, University of Gdańsk, Gdańsk, Poland; 2 Tri-City Animal Laboratory, Medical University of Gdańsk, Gdańsk, Poland; 3 Department of Medical Biotechnology, Intercollegiate Faculty of Biotechnology UG-MUG, Medical University of Gdańsk, Gdańsk, Poland; 4 Department of Microbiology, Faculty of Biology, University of Gdańsk, Gdańsk, Poland

**Keywords:** *Bacillus subtilis*, Recombinant spores, Adsorption, Mucosal immunization, FliD, *Clostridium difficile*

## Abstract

**Background:**

*Bacillus subtilis* spores can be used for presentation of heterologous proteins. Two main approaches have been developed, the recombinant one, requiring modification of bacterial genome to express a protein of interest as a fusion with spore-coat protein, and non-recombinant, based on the adsorption of a heterologous protein onto the spore. So far only single proteins have been displayed on the spore surface.

**Results:**

We have used a combined approach to adsorb and display FliD protein of *Clostridium difficile* on the surface of recombinant IL-2-presenting spores. Such spores presented FliD protein with efficiency comparable to FliD-adsorbed spores produced by wild-type 168 strain and elicited FliD-specific immune response in intranasally immunized mice.

**Conclusions:**

Our results indicate that such dual display technology may be useful in creation of spores simultaneously presenting adjuvant and antigen molecules. Regarding the characteristics of elicited immune response it seems plausible that such recombinant IL-2-presenting spores with adsorbed FliD protein might be an interesting candidate for vaccine against infections with *Clostridium difficile*.

**Electronic supplementary material:**

The online version of this article (doi:10.1186/s12934-017-0765-y) contains supplementary material, which is available to authorized users.

## Background

*Bacillus subtilis* spores are dormant forms of this microorganism, well known for their resistance to harsh environmental conditions. Their properties, combined with the easiness of genetic modification of this bacterium, make them a very convenient platform for presentation of heterologous proteins (reviewed in [1]). An interesting application of this technology is preparation of spore-based mucosal vaccines [2]. Use of such system enabled elicitation of protective immunity against infections with such pathogens as *C. perfringens* [3], *C. tetani* [4], *C. difficile* [5], or rotavirus [6].

There are two main approaches to display antigens on the surface of spores. Recombinant approach, developed as the first, is based on modification of bacterial genome in a way to express a protein of interest in fusion with one of spore coat proteins. As a result, a fusion protein is expressed in the cell and incorporated into the forming spore coat. Such method enables relatively simple construction of spores presenting heterologous proteins using basic methods of molecular biology. Non-recombinant approach is based on use of unmodified spores and adsorption of a purified protein of interest [7]. This method enables presentation of larger amounts of protein as compared to recombinant spores. Moreover, it does not lead to creation and use of GMO and thus can be much easier applied for animal or human use.

Spore-based vaccines can stimulate both systemic and localized immune responses with balanced Th1/Th2 polarization [8]. Although unmodified *B. subtilis* spores can be used as mucosal adjuvants in some applications [9] they can also be engineered to display immunomodulatory molecules and be administered as adjuvants in formulations with antigen-presenting spores [10]. It is worth noting, that no successful display of more than one recombinant protein on the surface of a single spore has been described in the literature. Such construct displaying molecules of both, an antigen and adjuvant, would be of special interest.

Following this line of reasoning, in this study we decided to use previously described recombinant spores presenting human IL-2 [10] and apply non-recombinant adsorption technique to display on their surface FliD flagellar cap protein of *Clostridium difficile*. The choice of antigen was not random, since *Clostridium difficile* is a well-known pathogen responsible for antibiotic-associated diarrheas and pseudomembranous colitis. Moreover, FliD protein possesses strong immunogenic properties [11, 12].

The results of performed immunization experiments suggest that such combined approach is promising and could be used for preparation of efficient spore-based formulations able to elicit antigen-specific immune responses with polarization driven by adjuvant presented on the surface of the same spore.

## Results

### Non-recombinant display of FliD protein

Our idea was to present on spore surface both, an adjuvant and antigen, therefore we decided to use recombinant spores produced by BKH121 strain, which display human IL-2 as fusion with CotB protein joined by a peptide linker. Single spore of this strain presents an average number of 9.5 × 10^4^ IL-2 molecules [10]. Since robust display of proteins on spore surface can be achieved using adsorption method [13–15] we also decided to apply this approach to present FliD protein of *C. difficile*. The entire FliD was overproduced in *E. coli*, purified and used for adsorption on surface of spores produced by the wild-type strain 168 and the recombinant strain BKH121.

We estimated amounts of spore-adsorbed FliD by measuring unbound protein in adsorption mixture and all subsequent washes (Additional file 1: Tables S1, S2). In the case of spores of both strains the results were comparable and reached levels of 4.13 × 10^4^ FliD molecules/spore and 3.66 × 10^4^ FliD molecules/spore for 168 and BKH121, respectively.

**Fig. 1:**
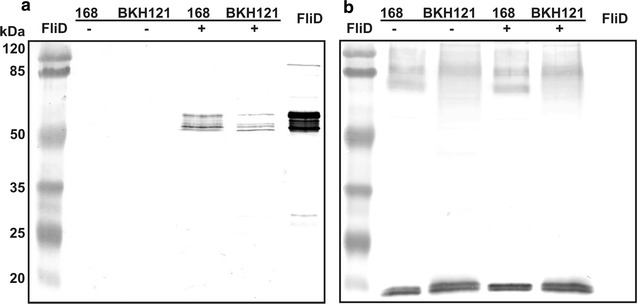
**a** Western blotting analysis of spore adsorption with purified FliD protein. Upon adsorption with purified FliD spore surface proteins were extracted by SDS-DTT treatment, fractionated on SDS-PAGE and analyzed by Western blotting. Spore coat extracts were prepared with spores of wild-type strain 168 and recombinant BKH121 alone or with adsorption with FliD (indicated with − and +). Purified FliD (1 μg) was used as positive control. **b** The same volumes of spore coat extracts were simultaneously loaded onto the second SDS-PAGE gel and analyzed by Western blotting using anti-CotZ antibodies to verify the equality of protein loading. The calculated molecular mass of CotZ is 16.4 kDa

**Fig. 2:**
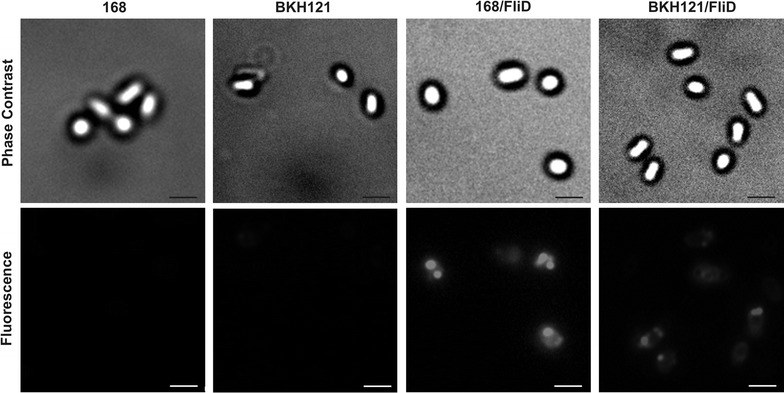
Spore surface display of adsorbed FliD protein as assessed by immunofluorescence microscopy. Purified, free spores of wild-type strain 168 and recombinant BKH121 alone and with adsorption of FliD protein (168/FliD, BKH121/FliD) were visualized by phase contrast and immunofluorescence microscopy. The spores were incubated with mouse anti-FliD antibodies, followed by anti-mouse IgG–Cy3 conjugates. The same exposure time was used for all immunofluorescence images. Scale bar—10 μm

Western blotting analysis of spore coat extracts showed presence of 56 kDa band which reacted with anti-FliD antibodies (Fig. 1a) indicating presence of this protein adsorbed on the spores. To visualize surface exposition of adsorbed protein we made use of immunofluorescence microscopy. Upon incubation with anti-FliD primary antibodies and anti-mouse IgG–Cy3 we observed in microscope fluorescent signal around spores of both strains which were subjected to FliD adsorption procedure (Fig. 2). In both experiments signal observed for 168/FliD spores was stronger than in the case of BKH121/FliD spores. These observations are in agreement with increased amount of FliD adsorbed on spores of the wild-type strain 168 in comparison to spores produced by BKH121. This suggests that the surface of recombinant spores used in the study possess diminished adsorption capability.

### Intranasal immunization of animal leads to development of FliD-specific immune response

**Fig. 3:**
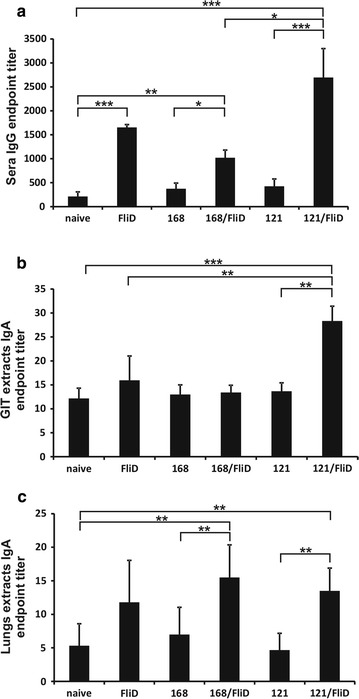
Antibody production in mice immunized with spore-adsorbed FliD. Groups (n = 6) of BALB/c mice were intranasally immunized with purified FliD, spores of alone (168, BKH121), or FliD-adsorbed spores (168/FliD, BKH121/FliD). **a** Anti-FliD IgG detected in mice serum at the end of treatment, anti-FliD IgA detected in saponin extracts of **b** gastrointestinal tracts, and **c** lungs of immunized animals. Antibody levels are expressed as endpoint titers. Error bars represent standard deviations. Statistical analysis performed as described in the “Methods” section. *P < 0.05; **P < 0.01; ***P < 0.001

Having verified the display of FliD protein on surface of spores, we used them for mucosal immunizations of mice applying two different administration routes: oral and intranasal. First immunization scheme (oral) consisted of a total of nine doses of 1 × 10^10^ spores delivering 39.2 μg of protein for FliD-adsorbed spores produced by 168 strain and 34.8 μg of protein for spores produced by BKH121 strain. In the case of this administration route we failed to observe eliciting FliD-specific immune response as assessed by production of antigen-specific antibodies in immunized animals (data not shown). Intranasal immunizations were performed by administration a total of eight doses of 5 × 10^9^ spores delivering 19.6 μg (168/FliD) and 17.4 μg (BKH121/FliD) of protein per dose. In the case of this administration route we detected FliD-specific IgG antibodies in sera the immunized animals. Increased levels of FliD-specific IgG antibodies were detected in sera of animals immunized with purified FliD protein and BKH121/FliD spores. The highest titers were observed for animals immunized with IL-2 presenting spores adsorbed with FliD protein (BKH121/FliD) (Fig. 3a). We also collected entire gastrointestinal tracts (GITs) and lungs of mice used in this experiment, and performed saponin extraction to assess levels of FliD-specific IgA antibodies in the analyzed material. We detected these antibodies in extracts prepared with both, lungs and GITs, with the highest levels observed in the case of mice immunized with FliD-adsorbed spores produced by the BKH121 (GIT extracts, Fig. 3b) and comparable levels in the case of both, 168/FliD and BKH121/FliD spores (lungs extracts, Fig. 3c). Immunization of mice with purified FliD protein did not significantly increase levels of IgA in both, GITs and lungs.

### Characterization of elicited immune response

**Fig. 4:**
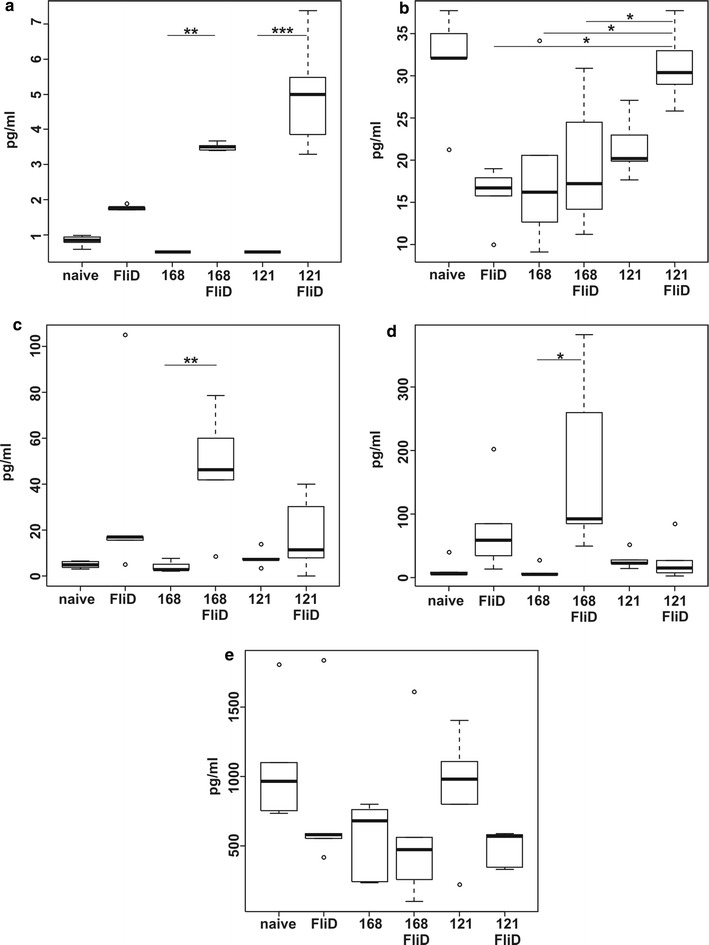
Characterization of the immune response. Groups (n = 6) of BALB/c mice were intranasally immunized with purified FliD, spores of alone (168, BKH121), or FliD-adsorbed spores (168/FliD, BKH121/FliD). Isolated splenocytes were stimulated with purified FliD and levels of following cytokines were measured in cell culture supernatants: **a** IL-2, **b** IL-10, **c** IL-17A, **d** IFN-γ, **e** TNF-α. Statistical analysis performed as described in the “Methods” section. *P < 0.05; **P < 0.01; ***P < 0.001

To characterize the polarization of immune response elicited by FliD-adsorbed spores we isolated spleens of immunized animals and used these organs to obtain splenocytes. Cells were stimulated with purified FliD protein and cell supernatants containing secreted cytokines were analyzed by flow cytometer using Cytometric Bead Array (CBA). With this method, we were able to measure levels of IL-2, IL-4, IL-6, IL-10, IL-17A, TNF-α and IFN-γ in single sample which enabled us to characterize polarization of observed immune response. We noticed no statistically significant changes in levels of TNF-α across all groups of immunized animals (Fig. 4e). IL-2 level was increased in supernatants of cells isolated from animals immunized with FliD-adsorbed spores of both, the wild type and BKH121 strains, as well as with purified FliD (Fig. 4a). The highest level of IL-2 was observed in samples corresponding to group immunized with BKH121/FliD spores. IL-10 level, interestingly, was high in the case of cells isolated from animals immunized with BKH121/FliD spores and comparable with cells isolated from naïve mice. In the case of other experimental groups we observed much lower level of this cytokine (Fig. 4b). IL-17A level showed statistically significant (*P* = 0.0004) increase in supernatants of cells isolates from animals immunized with 168/FliD spores. Some increase was observed in the case of samples corresponding to experimental groups immunized with either purified FliD protein or BKH121/FliD spores, nevertheless the increase was not statistically significant (Fig. 4c). We also noticed statistically significant (*P* = 0.0002) increase in the level of IFN-γ in supernatant of cells isolated from animals immunized with 168/FliD spores (Fig. 4d). We were not able to detect IL-4 and IL-6 in any of analyzed samples.

### Serum IgG response to native FliD in *Clostridium difficile*

**Fig. 5:**
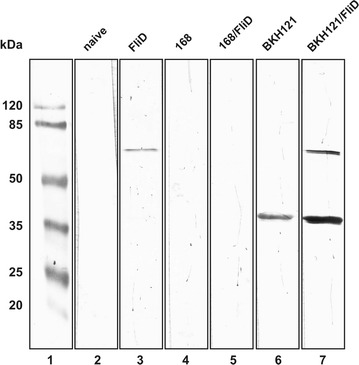
Detection of native FliD of *Clostridium difficile* 630 by sera of immunized animals. Flagellar preparations were fractionated on SDS-PAGE and analyzed by Western blotting with pooled sera of each group of immunized animals. The lines 3–7 correspond to groups of animals administered with: (3) purified FliD, (4) empty 168 spores, (5) 168/FliD spores, (6) empty BKH121 spores, (7) BKH121/FliD spores. (1) Molecular mass marker, (2) the naïve group

To verify whether sera of mice immunized with FliD-adsorbed spores recognize native FliD in *Clostridium difficile* we used pooled sera of each experimental group in Western blotting of *C. difficile* 630 flagellar preparations. We observed a specific band corresponding to 56 kDa (a calculated molecular mass of FliD protein) in the case of sera of animals immunized with purified FliD (Fig. 5 lane 3) and a visibly stronger band of the same molecular mass in the case of sera of animals immunized with BKH121/FliD spores (Fig. 5 lane 7). Interestingly, we observed another band corresponding to a molecular mass of approximately 40 kDa in the case of sera of groups administrated with BKH121 (Fig. 5 lane 6) and BKH121/FliD (Fig. 5 lane 7) spores. Since this band seemed to be specific for immunizations with IL-2-presenting spores we performed BLAST analysis [16, 17] of amino acid sequences of IL-2 fragment and a short peptide linker (SGGGEAAAKGGG) attaching IL-2 to CotB protein in BKH121 spores against the database of *C. difficile* 630 protein sequences. We filtered out the BLAST results for proteins characterized as extracellular [18] and found nine (Additional file 1: Table S3), out of which two possessed a molecular mass close to the mass corresponding the observed band. These proteins were a flagellar hook-associated protein FlgK (47.9 kDa) and an elongation factor Tu (43.8 kDa). Obtained results indicate that the immunization of mice with spores adsorbed with purified FliD led to production of FliD-specific IgG antibodies in sera of these animals, capable of recognizing the native FliD of *C. difficile*. Moreover, the eliciting of the efficient immune response required presence of IL-2, which served as an adjuvant.

## Discussion

The technology of spore display has been used in different applications [1]. One of the most interesting is the use of *B. subtilis* spores as carriers of antigens in mucosal vaccines. It is important to note, that no studies have been described in the literature which would involve creation of recombinant spores displaying more than a single protein. In our work we have overcome this issue by applying a combination of both approaches to spore display, recombinant and non-recombinant.

While the use of recombinant spores is well described in the literature [1, 2, 19], the non-recombinant approach seems to be becoming an attractive alternative, enabling much more efficient display of proteins on the spore surface [7]. Following this idea we used adsorption method to display entire FliD protein on the spores. The efficiency of display was 8–48 times higher as compared to recombinant spores displaying FliD fragment in fusion with spore coat proteins, constructed in one of our previous studies [20]. Interestingly, spores produced by the wild-type 168 strain adsorbed on their surface slightly more FliD protein than spores of the isogenic recombinant strain BKH121 (Additional file 1: Tables S1, S2). Observed nearly 12% difference is most probably the consequence of altered spore surface due to the presentation of IL-2 attached to CotB protein via a peptide linker. It is also worth noting that the overall staining pattern in the immunofluorescence microscopy seems to be punctate rather than uniform (Fig. 2), which might suggest that FliD protein penetrates deeper into the spore coat structure. As recently reported, such phenomenon might result in lowered binding of antibodies directed against adsorbed protein and hence altered staining pattern [21].

From our previous studies we knew that BKH121 spores co-administrated by oral route with other recombinant spores presenting antigen worked as adjuvant and helped in development of antigen-specific immune response [10]. In current research lack of FliD-specific antibodies produced upon oral immunization of animals suggests that non-recombinant spores presenting FliD are not compatible with this route of administration. Since FliD-adsorbed spores deliver large amounts of this protein, the lack of elicited immune response might be the result of development of tolerance to the antigen. Another explanation is that FliD protein is released from the spore surface and degraded in stomach environment. To overcome this problem we decided to use an alternative, intranasal route. Such approach has also been used by other research groups which applied intranasal route to immunize mice with non-recombinant spores presenting influenza H5N1 virions [9] and *Mycobacterium tuberculosis* antigens [22]. It is important to emphasize, that intranasal route of antigen administration can confer protection against enteric pathogens. Intranasal immunization of mice with *Neospora caninum* (a protozoan infecting the GIT of a wide range of mammalian hosts) antigen extracts results in establishing long-term protection and long-lasting elevated levels of parasite-specific serum IgG and intestinal IgA [23].

In our study intranasal administration of non-recombinant spores displaying FliD resulted in production of FliD-specific IgG antibodies in sera of immunized animals (Fig. 3a). This observation corresponds with high immunogenicity of this protein [11, 12]. Moreover, lower titers of FliD-specific IgG antibodies in sera of animals immunized with purified FliD protein emphasizes usefulness of spore adsorption technology for the delivery of antigens in mucosal vaccines. As we expected, the highest titers were observed after administration of recombinant spores displaying IL-2 with adsorbed FliD protein. What was important, the sera of animals immunized with either these spores or purified FliD recognized native *Clostridium difficile* 630 FliD protein in flagellar preparations (Fig. 5). Moreover, the signal observed in the case of sera of animals immunized with BKH121/FliD spores was stronger. Taken together these observations confirm the adjuvant action of IL-2 present on the surface of these spores. It is worth note, that although results of ELISA experiments showed statistically significant increase in the level of FliD-specific IgG antibodies in sera of animal immunized with 168/FliD spores (Fig. 3a), it was most probably too low to detect native FliD of *C. difficile* by the Western blotting technique (Fig. 5 lane 5). Interestingly, the immunization with BKH121 spores leads to the production of IgG antibodies recognizing another antigen of *C. difficile*, with the molecular mass close to 40 kDa, present in the flagellar preparation (Fig. 5 lanes 6 and 7). Since no such band was observed in the Western blotting performed with sera of animals immunized with spores produced by the wild-type 168 strain, either IL-2 fragment present in the BKH121 spores or a peptide linker attaching this protein to the CotB is responsible for development of these antibodies specific for *C. difficile* antigen.

A trend similar to the one observed in the case of sera IgG antibodies was noticed for FliD-specific IgA antibodies extracted from entire GITs of immunized animals (Fig. 3b). This time BKH121/FliD spores were the only to result in of significantly increased production of these antibodies. Interestingly, while administration of FliD-adsorbed spores produced by both, the wild-type 168 and BKH121 strains resulted in production of statistically significant levels of FliD-specific IgA antibodies in lungs of immunized animals, the highest levels of these antibodies were observed in groups immunized with 168/FliD spores (Fig. 3c). This observation might be surprising, because co-administration [24] or co-expression of IL-2 in microorganisms used for immunization of animals [25, 26] led to increased production of antigen-specific secretory IgA antibodies in lungs. Nevertheless, it is difficult to confront these findings with our observations because of use of different antigens, antigen-delivery systems and lack of information regarding amounts of IL-2 used in cited works.

The polarization of elicited immune response, as assessed by the analysis of cytokines produced by FliD-sensitized splenocytes, confirms the assumption of different immunomodulation caused by the presence of IL-2 on surface of BKH121 spores. FliD-presenting spores of the wild type strain led to bias of the response towards Th1 and Th17. Such polarization has also been observed in our previous studies involving use of recombinant spores presenting FliD fragment [27, 28]. Lack of IL-6 (data not shown), a cytokine characteristic for Th17 polarization of the immune response, seems to result from technical problems in detecting this cytokine with the method used in our study. Intranasal administration of BKH121/FliD spores resulted in production of IL-2 by FliD-sensitized splenocytes (Fig. 4a), which is one of hallmarks of Th1 polarization. Observed production of IL-10 (Fig. 4b) could generally be connected with Th2 polarization of elicited immune response, nevertheless in the case of mucosal immunity this cytokine is thought to be produced mainly by T_R_ cells [29]. Hence, in our experiments with BKH121/FliD spores we most probably observe moderate Th1 polarization (lack of typical Th1 cytokines i.e. IFN-γ, TNF-α) accompanied by the activity of T_R_ cells. Taken these facts together with observed production of FliD-specific IgA antibodies in GITs of immunized animals (Fig. 3b) we can assume, that spores with a combined recombinant display of IL-2 and non-recombinant display of FliD may be useful in immunization against infections with *Clostridium difficile*. This assumption is supported by the fact, that the immune homeostasis of intestines, constantly exposed to antigens of commensal microflora, is thought to be maintained due to the balance of high levels of T_R_ and low levels of Th17 cells, accompanied by the production of anti-inflammatory cytokines IL-10 and TGF-β [30]. Intense antibiotic treatment, which precedes outbreak of *C. difficile* infection, causes disturbance of natural intestinal microflora and leads to decrease in levels of T_R_ in favor of Th17 cells. This coupled with the action of clostridial toxins perpetuates a pro-inflammatory response (reviewed in [31]). It also important to note, that IgA antibodies play a critical role in mucosal immunity and therefore should be important in protection against *C. difficile* [32, 33].

Overall pattern of FliD-specific antibodies production in the immunized animals, as well as the polarization of elicited immune response justifies the hypothesis that BKH121/FliD spores enhance production of IL-2 and IL-10, which are known to be stimulating intestinal IgA class switching [34]. This could lead to the increase of FliD-specific IgA levels in GITs of immunized animals. On the other hand, the levels of these cytokines produced in the animals immunized with 168/FliD spores is most probably too low to efficiently trigger production of FliD-specific IgA in such distant location as the GIT, limiting it to the local response in the respiratory tract.

FliD protein has also been used before for immunization of mice in the work of Péchiné et al. [35]. In that study the intranasal immunization with FliD-containing microparticles resulted in elevated levels of FliD-specific serum IgG antibodies with no statistically significant levels of FliD-specific IgA antibodies detected in the intestinal lavages of immunized animals. Nevertheless, it is difficult to directly compare these results with the results of our study, because of differences in FliD preparation for immunization (microparticles vs. purified protein), differences in antigen administration (in the work of Pechine et al. intranasal administration of FliD-containing microparticles was performed along with cholera toxin), different immunization schemes and other animals used in the research (Balb/c vs. C3H).

## Conclusions

The results of our study show that a combined recombinant and non-recombinant approach to presentation of proteins on surface of *B. subtilis* spores is promising and enables simultaneous display of two types of molecules exhibiting distinct activities. Spores simultaneously presenting adjuvant and antigen are good example of application of this technology. In our study these were spores displaying IL-2 and *C. difficile* FliD which also seem to be interesting candidates for vaccine against infections with this pathogen.

## Methods

### Bacterial strains

*Bacillus subtilis* strains 168 [36] and BKH121 (CotB-IL-2) [10], and *Clostridium difficile* 630 [37] were used in this study.

### Preparation of *Bacillus subtilis spores*

Sporulation was induced by the exhaustion method in DS (Difco-Sporulation) medium as described elsewhere [38]. Sporulating cultures were harvested 24 h after the initiation of sporulation and purified using a lysozyme treatment to break up any residual sporangial cells, followed by washing steps in 1 M NaCl, 1 M KCl and water (twice each), as previously described [18]. PMSF (0.05 M) was included to inhibit proteolysis. After the final suspension in water spores were treated at 65 °C for 1 h to kill any residual vegetative cells. The spore suspension was titrated immediately for CFU/ml before freezing at −22 °C. By this method we could reliably produce 6 × 10^10^ spores per liter of DSM culture.

### Adsorption of FliD protein onto the spores

100 μg of purified FliD [20] was added to a suspension of 3 × 10^10^ spores in 0.15 M PBS pH 4.0 at room temperature in a total volume of 1.5 ml. After 1 h of incubation spores suspension was centrifuged (10 min at 13,000×*g*) and washed three times with 0.15 M PBS pH 7.2. The final pellet was resuspended in 120 μl of 0.15 M PBS pH 7.2.

### Visualization of spore-adsorbed FliD by Western blotting

1 × 10^8^ spores were resuspended in 100 μl of water and centrifuged for 10 min at 11,000×*g*. Pellet was resuspended in 30 μl of spore coat extraction buffer (50 mM Tris–HCL pH = 6.8, 1% SDS, 50 mM DTT) and incubated with shaking (150–300 rpm) for 1 h at 65 °C to solubilize spore coat proteins. Upon centrifugation (10 min, 11,000×*g*) supernatant was loaded onto a 12% SDS-PAGE gel. The proteins were then electrotransferred to nitrocellulose filters (Life Technologies) and used for Western blotting analysis as previously described [20]. Anti-CotZ antiserum was used for verification of the equality of protein loading.

### Quantification of spore-adsorbed FliD

Supernatants of adsorption mixture and washings was used for ELISA assay to assess the amount of unbound FliD. 96-well Maxisporp ELISA plates (NUNC) were coated overnight at 4 °C with collected supernatants. Wells were blocked with 0.5% BSA in PBS for 1 h 37 °C at room temperature. Plates were incubated for 2 h with primary mouse anti-FliD antibodies (Negri, 2013) at 37 °C and then for 1 h with secondary anti-mouse IgG conjugated with HRP (Sigma). After incubations 1 mg/ml of o-phenylenediamine dihydrochloride prepared from SIGMAFAST Tablets (Sigma) was added as a substrate for HRP. The reaction was stopped with 3 M H_2_SO_4_ and absorbance was read at 492 nm. Purified FliD was used to prepare calibration curve. The amounts of FliD measured in adsorption mixture and three steps of washing were summed up to obtain a total of unbound FliD. The amount of protein adsorbed onto the spores was calculated by subtraction of the amount of unbound FliD from the amount of FliD used in the adsorption mixture, and recalculated to number of FliD molecules per single spore.

### Immunofluorescence microscopy

Staining of spores with mouse anti-FliD or rabbit anti-IL-2 (Abcam) antibodies and immunofluorescence microscopy were performed as previously described [20]. Spores were blocked with 3% skimmed milk in PBS for 30 min at room temperature and washing another three times in PBS. Samples were separately incubated overnight at 4 °C with anti–FliD or anti-IL-2 antibodies, washed three times and then incubated with anti-mouse Cy3-conjugated IgG and anti-rabbit FITC-conjugated IgG (Jackson ImmunoResearch Laboratories) overnight at 4 °C. After three washes with PBS, samples were loaded on microscope slides previously coated with poly-l-lysine (Sigma). The coverslips were mounted onto a microscope slide and viewed using a Zeiss Axioplan fluorescence microscope with the same exposure time for all samples. Images were captured using a camera connected to the microscope, processed with Corel Photo-Paint software and saved in TIFF format.

### Immunizations

Five groups of six mice (female, BALB/c, 8 weeks) were immunized by oral or intranasal route with suspensions of either spores of the wild-type strain 168 with adsorbed FliD protein, empty spores of 168, BKH121 spores with adsorbed FliD, empty spores of BKH121, or 2 μg of purified FliD protein per single dose. A naive, non-immunized control group was included. Oral immunizations were performed with 1.0 × 10^10^ spores in a volume of 0.2 ml of water administered by intragastric gavage on days 1, 3, 5, 22, 24, 26, 43, 45, 47. Animals were killed on day 61 and serum samples, spleens and gastrointestinal tracts were collected. Intranasal immunizations were performed with 5 × 10^9^ spores in volume of 20 μl of water administered once a week for 8 weeks [39]. Upon completion of immunization cycle animals were killed and serum samples, spleens, lungs and gastrointestinal tracts were collected.

### Indirect ELISA for detection of antigen-specific antibodies

FliD-specific antibodies in saponin extracts of gastrointestinal tracts or lungs, and sera of immunized animals were detected as previously described [10]. Briefly, plates were coated with 100 μl per well of the specific antigen (2 μg/ml in carbonate/bicarbonate buffer) and left at room temperature overnight. Purified FliD protein was used as antigen. After blocking with 0.5% BSA in PBS for 1 h at 37 °C samples were applied using a twofold dilution series in ELISA diluent buffer (0.1 M Tris–HCl, pH 7.4; 3% (w/v) NaCl; 0.5% (w/v) BSA; 10% (v/v) sheep serum (Sigma); 0.1% (v/v) Triton-X-100; 0.05% (v/v) Tween-20). Every plate carried replicate wells of a negative control (a 1/20 diluted pre-immune serum), and a positive control prepared with serial dilutions of control serum from mice immunized intraperitoneally with FliD purified protein. Plates were incubated for 2 h at 37 °C before addition of anti-mouse AP conjugates (Sigma). Plates were incubated for a further 1 h at 37 °C then developed using the substrate pNPP (para-Nitrophenylphosphate; Sigma). Reactions were stopped using 2 M H_2_SO_4_ and absorbance was read at 450 nm. The results were expressed as reciprocal endpoint titer of the last dilution exhibiting an O.D. equal or greater than O.D. of negative controls increased by one their standard deviation.

### Isolation of splenocytes

Mice were killed and spleens were isolated as previously described [10]. The spleens were perfused with RPMI-1640 (supplemented with 10% heat inactivated fetal calf serum, 25 mM HEPES, 2 mM l-glutamine, 1 mM sodium pyruvate, 100 IU/ml penicillin and 100 mg/ml streptomycin) using 5 ml syringe fitted with 26 G needle to obtain single cell suspension of splenocytes. The splenocytes suspension was then centrifuged at 300×*g* for 15 min. The RBCs were lysed by hypotonic shock using 3 ml of 0.84% of sterile NH_4_Cl or ACK lysis buffer for 5 min. The cells were then washed thrice with RPMI-1640 to remove lysed RBCs and NH_4_Cl.

### Activation of splenocytes

Splenocytes (2 × 10^5^/ml) were cultured in presence or absence of FliD antigen for 48 h. Samples of supernatants containing released cytokines were collected and stored at −80 °C.

### Measurement of released cytokines

Levels of IL-10, IL-17, TNF, IFN-γ IL-6, IL-4 and IL-2 secreted by sensitized cells were determined by Cytometric Bead Array (CBA) Mouse Th1/Th2/Th17 Cytokine Kit (BD) kit according to manufacturer’s protocol. Measurements were performed using Accuri C6 Flow Cytometer (BD) and the results processed with system software. Six technical repeats were done for each animal in the group.

### Partial *Clostridium difficile* flagella purification

Partial *C. difficile* flagella purification was performed as previously described [40] with following modifications. Bacteria were grown overnight in BHI medium supplemented with 0.5% yeast extract and 0.1% l-cysteine at 37 °C under anaerobic conditions. Bacterial cells were pelleted by centrifugation at 5000×*g* for 15 min at 4 °C. The pellets were resuspended in 5 ml of distilled water, strongly vortexed for 1 min and centrifuged at 5000×*g* for 30 min at 4 °C. The supernatants were centrifuged at 25,000×*g* for 1 h at 4 °C. The pellets were suspended in 100 μl of phosphate-buffered saline (PBS) (pH 7.4).

### Recognition of *C. difficile* FliD by sera of immunized animals

3 volumes of flagella preparation were mixed with 1 volume of 4x SDS-PAGE sample buffer (62.5 mM Tris–HCl pH 6.8, 10% glycerol, 1% SDS, 0.005% bromophenol blue), incubated for 5 min at 100 °C and loaded onto 12% SDS-PAGE gel. Separated proteins were then electrotransferred to nitrocellulose filter (Life Technologies) which was then cut into individual lanes and blocked for 1 h in TBS-Tween 20 buffer containing 3% skimmed milk. Slices of the filter were incubated overnight in 1:25 dilutions of sera in 3% skimmed milk/TBS-Tween 20 pooled for each group of immunized animals. Further processing of the filters was performed as previously described [20].

### Statistical analysis

Differences among the various experimental groups were determined by the Kruskal–Wallis non-parametric test, followed by Dunn’s Multiple Comparison Test for post hoc analysis.

## Additional file

**Additional file 1: Tables S1 and S2.** ELISA quantification of the FliD adsorption reaction performed with wild-type 168 and BKH121 spores. **Table S3.** Extracellular proteins sharing sequence homology with IL2-fragment and a peptide linker present in the BKH121 spores as assessed by the BLAST analysis.
