# Supplementary material for: The combination of recombinant and non-recombinant Bacillus subtilis spore display technology for presentation of antigen and adjuvant on single spore
Source: Microb Cell Fact. 2017 Sep 12;16:151. doi: 10.1186/s12934-017-0765-y (PMC5596941; doi:10.1186/s12934-017-0765-y)
Supplement: Supplementary file 1 — Additional file 1: Tables S1 and S2. ELISA quantification of the FliD adsorption reaction performed with wild-type 168 and BKH121 spores. Table S3. Extracellular proteins sharing sequence homology with IL2-fragment and a peptide linker present in the BKH121 spores as assessed by the BLAST analysis. [file 12934_2017_765_MOESM1_ESM.pdf]

Table S1. ELISA quantification of FliD protein adsorbed on the spores produced by wild-type 168 strain.

**Purified FliD**		**Adsorption supernatant**			**Wash I**			**Wash II**			**Wash III**		
ng	OD_{492}	µl	OD_{492}	FliD ng	µl	OD_{492}	FliD ng	µl	OD_{492}	FliD ng	µl	OD_{492}	FliD ng
10	0.881	10	NA	NA	10	0.836	9.3	10	0.127	1.3	10	0.191	2.0
5	0.484	5	1.387	14.5	5	0.513	5.6	5	0.051	0.5	5	0.091	0.9
2.5	0.217	2.5	0.747	7.8	2.5	0.270	2.9	2.5	0.023	0.2	2.5	0.051	0.5
1.25	0.112	1.25	0.351	3.7	1.25	0.135	1.4	1.25	0.009	0.1	1.25	0.022	0.2
0.62	0.066	0.62	0.179	1.9	0.62	0.065	0.6	0.62	NA	NA	0.62	0.009	0.1
0.31	0.030	0.31	0.092	0.9	0.31	0.032	0.3	0.31	NA	NA	0.31	NA	NA
0.15	0.017	0.15	0.044	0.5	0.15	0.015	0.1	0.15	NA	NA	0.15	NA	NA
			**Unbound FliD**	**4621.4**			**4818.3**			**459.5**			**821.8**
		**FliD used for adsorption**			**50.0 µg**								
		**Total unbound FliD**			**10.7 µg**		**Number of FliD**						
		**Total bound FliD/1x10^{10} spores**			**39.3 µg**		**molecules/spore**		**4.1x10^{4}**				

Table S2. ELISA quantification of FliD protein adsorbed on the spores produced by BKH121 strain.

**Purified FliD**		**Adsorption supernatant**			**Wash I**			**Wash II**			**Wash III**		
ng	OD_{492}	µl	OD_{492}	FliD ng	µl	OD_{492}	FliD ng	µl	OD_{492}	FliD ng	µl	OD_{492}	FliD ng
10	0.455	10	0.838	18.3	10	0.836	18.3	10	0.127	2.7	100	0.191	4.2
5	0.226	5	0.468	10.2	5	0.513	11.2	5	0.051	1.1	50	0.091	1.9
2.5	0.124	2.5	0.262	5.7	2.5	0.270	5.9	2.5	0.023	0.5	25	0.051	1.1
1.25	0.058	1.25	0.122	2.6	1.25	0.135	2.9	1.25	0.009	0.2	12.5	0.022	0.5
0.62	0.028	0.62	0.053	1.1	0.62	0.065	1.4	0.62	NA	NA	6.25	0.009	0.2
0.31	0.014	0.31	0.024	0.5	0.31	0.032	0.6	0.31	NA	NA	3.12	NA	NA
0.15	0.004	0.15	0.014	0.3	0.15	0.015	0.3	0.15	NA	NA	1.56	NA	NA
			**Unbound FliD**	**3060.4**			**10831.8**			**1064.5**			**207.1**
		**FliD used for adsorption**			**50.0 µg**								
		**Total unbound FliD**			**15.1 µg**		**Number of FliD**						
		**Total bound FliD/1x10^{10} spores**			**34.9 µg**		**molecules/spore**		**3.6x10^{4}**				

Table S3. Extracellular proteins sharing sequence homology with IL2-fragment and a peptide linker present in the BKH121 spores as assessed by the BLAST analysis

**Locus tag**	**Annotation**	**Molecular mass kDa**	**Query**	**BLAST search strategy**
CD630_05140	hemagglutinin/adhesion (CwpV)	166.5	linker-IL-2	PSI-BLAST
CD630_14690	cell surface protein penicillin-binding protein (Cwp20)	111.0	linker	DELTA-BLAST
CD630_27840	N-acetylmuramoyl-L-alanine amidase, autolysin (Cwp6)	72.9	linker	DELTA-BLAST
CD630_27910	cell wall binding protein (Cwp12)	66.3	linker	DELTA-BLAST
CD630_27900	LmbE-like deacetylase	26.8	linker	DELTA-BLAST
CD630_02310	flagellar hook-associated protein FlgK (HAP1)	47.9	linker-IL-2	PSI-BLAST
CD630_02680	flagellar basal body rod protein FlgG	28.0	linker	DELTA-BLAST
CD630_00580	elongation factor Tu (EF-Tu)	43.9	linker-IL-2	blastp
CD630_31470	glyceraldehyde-3-phosphate dehydrogenase (GAPDH)	62.5	linker-IL-2	PSI-BLAST
